# A matter of age? How age affects the adaptation of lactating dairy cows to virtual fencing

**DOI:** 10.1093/jas/skae137

**Published:** 2024-05-14

**Authors:** Andrea Confessore, Manuel K Schneider, Caren M Pauler, Chiara Aquilani, Patricia Fuchs, Carolina Pugliese, Camilla Dibari, Giovanni Argenti, Pier Attilio Accorsi, Massimiliano Probo

**Affiliations:** Department of Agriculture, Food, Environment, and Forestry (DAGRI), Università di Firenze, Via delle Cascine 5, Firenze, 50144, FI, Italy; Agroscope, Research Division Animal Production Systems and Animal Healt, Forage Production and Grassland Systems, 8046 Zurich, Switzerland; Agroscope, Research Division Animal Production Systems and Animal Healt, Grazing Systems, 1725 Posieux, Switzerland; Agroscope, Research Division Animal Production Systems and Animal Healt, Forage Production and Grassland Systems, 8046 Zurich, Switzerland; Agroscope, Research Division Animal Production Systems and Animal Healt, Grazing Systems, 1725 Posieux, Switzerland; Department of Agriculture, Food, Environment, and Forestry (DAGRI), Università di Firenze, Via delle Cascine 5, Firenze, 50144, FI, Italy; Graduate School for Cellular and Biomedical Sciences, University of Bern, 3012 Bern, Switzerland; Agroscope, Research Division Animal Production Systems and Animal Healt, Grazing Systems, 1725 Posieux, Switzerland; Department of Agriculture, Food, Environment, and Forestry (DAGRI), Università di Firenze, Via delle Cascine 5, Firenze, 50144, FI, Italy; Department of Agriculture, Food, Environment, and Forestry (DAGRI), Università di Firenze, Via delle Cascine 5, Firenze, 50144, FI, Italy; Department of Agriculture, Food, Environment, and Forestry (DAGRI), Università di Firenze, Via delle Cascine 5, Firenze, 50144, FI, Italy; Dipartimento di Scienze Mediche Veterinarie, Università di Bologna, Ozzano Emilia, 40064, BO, Italy; Agroscope, Research Division Animal Production Systems and Animal Healt, Grazing Systems, 1725 Posieux, Switzerland

**Keywords:** animal welfare, herd management, Holstein cattle, lactating cows, precision livestock farming, stress

## Abstract

Virtual Fencing (**VF**) can be a helpful technology in managing herds in pasture-based systems. In VF systems, animals wear a VF collar using global positioning, and physical boundaries are replaced by virtual ones. The Nofence (Nofence AS, Batnfjordsøra, Norway) collars used in this study emit an acoustic warning when an animal approaches the virtual boundaries, followed by an aversive electrical pulse if the animal does not return to the defined area. The stimuli sequence is repeated up to three times if the animal continues to walk forward. Although it has been demonstrated that animals successfully learn to adapt to the system, it is unknown if this adaptation changes with animal age and thus has consequences for VF training and animal welfare. This study compared the ability of younger and older dairy cows to adapt to a VF system and whether age affected activity behavior, milk yield, and animal long-term stress under VF management. The study was conducted on four comparable strip-grazing paddocks. Twenty lactating Holstein-Friesian cows, divided into four groups of five animals each, were equipped with VF collars and pedometers. Groups differed in age: two groups of older cows (>4 lactations) and two groups of younger ones (first lactation). After a 7-d training, paddock sizes were increased by successively moving the virtual fence during four consecutive grazing periods. Throughout the study, the pedometers recorded daily step count, time spent standing, and time spent lying. For the determination of long-term stress, hair samples were collected on the first and last day of the trial and the hair cortisol content was assessed. Data were analyzed by generalized mixed-effect models. Overall, age had no significant impact on animal responses to VF, but there were interaction effects of time: the number of acoustic warnings in the last period was higher in younger cows (*P* < 0.001), and the duration of acoustic warnings at training was shorter for older cows (*P* < 0.01). Moreover, younger cows walked more per day during the training (*P* < 0.01). Finally, no effects on milk yield or hair cortisol content were detected. In conclusion, all cows, regardless of age, adapted rapidly to the VF system without compromising their welfare according to the indicators measured.

## Teaser

This study demonstrates that old lactating dairy cows learn a virtual fencing system as fast as young lactating dairy cows, without affecting their activity behavior or causing long-term stress responses.

## Introduction

Improving the efficiency of grazing management is crucial to dairy farmers, not only to support herd requirements but also to reach high milk quality standards (Wilkinson et al., 2020). For instance, new and frequent pasture allocations promote milk production in dairy cows (Abrahamse et al., 2008). However, building, maintaining, and moving fences on pastures is time-consuming and therefore expensive. Technical innovations replacing physical fences have the potential to increase the positive outcomes of pasture-based systems in terms of herd management, grassland conservation, and animal welfare (Aquilani et al., 2022). Virtual Fencing (**VF**) systems represent one of the most promising technologies for achieving these objectives (Waterhouse, 2023). The VF replaces physical fences with virtual ones defined in a geographic information system environment only. There are currently four commercial VF systems with similar characteristics and capabilities (Goliński et al., 2023): in general, each animal wears a VF collar that uses global positioning to monitor their distance to the pre-set virtual boundary. When the animal crosses this virtual boundary, the VF collar emits an acoustic warning. If the animal continues to walk forward, the collar emits an aversive stimulus (i.e., a mild electric pulse, a vibration, or a combination of both—depending on the commercial system). In the application of this technology, there are two major concerns, namely the animal’s ability in learning to adapt to the system and the impacts on animal welfare (Stampa et al., 2020). Several studies have demonstrated that animals learn to interpret the acoustic warning correctly within 2 d and thus can avoid the electrical pulse, irrespective of being tested individually (Campbell et al., 2018) or in groups (Colusso et al., 2020). For the latter, this also might rely on the response of their herd mates, rather than directly receiving stimuli themselves (Keshavarzi et al., 2020). For instance, Lomax et al. (2019) showed that a group of 12 non-lactating Holstein-Friesian cows stayed within their assigned grazing areas 99% of the time, depicting a decreasing mean number of daily electrical pulses. An experiment by Verdon et al. (2021) observed comparable effectiveness of VF to electric fencing in keeping lactating cows within a predefined area, without affecting cow behavior, welfare, and milk yield. Similar results were obtained in other studies conducted on sheep (Marini et al., 2018; Campbell et al., 2021) and both cosmopolitan (Campbell et al., 2017; Confessore et al., 2022b; Fuchs et al., 2024) and autochthonous (Confessore et al., 2022a) beef cattle breeds. The preceding acoustic signal makes the electrical pulse highly predictable and controllable for the animals. Consistently, no evidence of long-term stress was found in previous studies (Lee et al., 2018; Kearton et al., 2020).

The age of cattle tested in previous VF experiments varied extremely: from very young (i.e., 3 to 6 mo old) to old animals (i.e., 6 to 9 yr old). However, there is no clear evidence on how age influences the learning process and the adaptations of grazing cows to a VF system. It is well known that aging leads to a decline in the cognitive abilities of humans (Raz et al., 2000; Seidler, 2006). At the same time, a study conducted on cattle (Kovalčik and Kovalčik, 1986)—not related to VF—showed that younger animals have a higher learning capacity than older cows (i.e., 15-mo-old heifers vs. cows at first lactation vs. cows after second lactation), but a less stable long-term memory. Similarly, Jago et al. (2011) found that heifers adapted more quickly than cows to a pasture-based automated milking system. Despite this, only one study considered the effect of age on adaption to VF in cattle (Verdon and Rawnsley, 2020). In that study, dairy heifers close to the calving age (i.e., 22 mo old), trained in an individual 5-d feed attractant trial, showed a faster adaptation to a VF system than heifers trained during an early age (i.e., < 12 mo old). However, the differences among age groups were very small, probably due to the small difference in age. The assumption that age may influence learning behavior in a VF system is underlined by the fact that many other aspects of dairy farming are affected by cattle age. For instance, age-dependent factors affect cow lifetime production (Haworth et al., 2008; Boothby et al., 2020), retention of early pregnancy (Starbuck et al., 2004), as well as feeding, ruminating, and digestion characteristics (Grandl et al., 2016). Furthermore, social foraging behavior is affected by animal age, with older and larger cattle being dominant during grazing (Sahu et al., 2020; Deniz et al., 2021).

The present study investigates the differences in the learning ability of younger and older lactating Holstein-Friesian cows managed under a strip-grazing system with VF. We hypothesized that younger dairy cows learn to adapt to a VF system faster than older dairy cows. Thus, we expected the younger animals to 1) have a faster increase in their success rate 2) receive a lower number of electrical pulses, 3) have lower long-term stress assessed in hair cortisol content, as well as 4) show a less potential depression in milk yield compared to older animals.

## Material and Methods

### Study area and environmental conditions

All experimental procedures were conducted according to the Swiss guidelines for animal welfare and were approved by the Animal Care Committee of the Canton of Fribourg, Switzerland (license 34580_2022-07-FR). The experiment took place from June to July 2022 during 31 d in the Swiss lowlands at the Agroscope experimental Institute in Posieux (46° 45ʹ 59.0″ N, 7° 6ʹ 17.2″ E). Mean daily Temperature Humidity Index (**THI**) was calculated as described in Ravagnolo et al. (2000). During the trial, precipitation sum was 158 mm and several heat waves occurred, resulting in a THI averaged (mean ± SD) of 68.93 ± 8.09. In addition, the average length of sunlight and twilight were 15 h and 1 h 20 min, respectively. While the average time of sunrise was at ⁓0540 hours local time.

### Animals and housing

**Figure 1.**
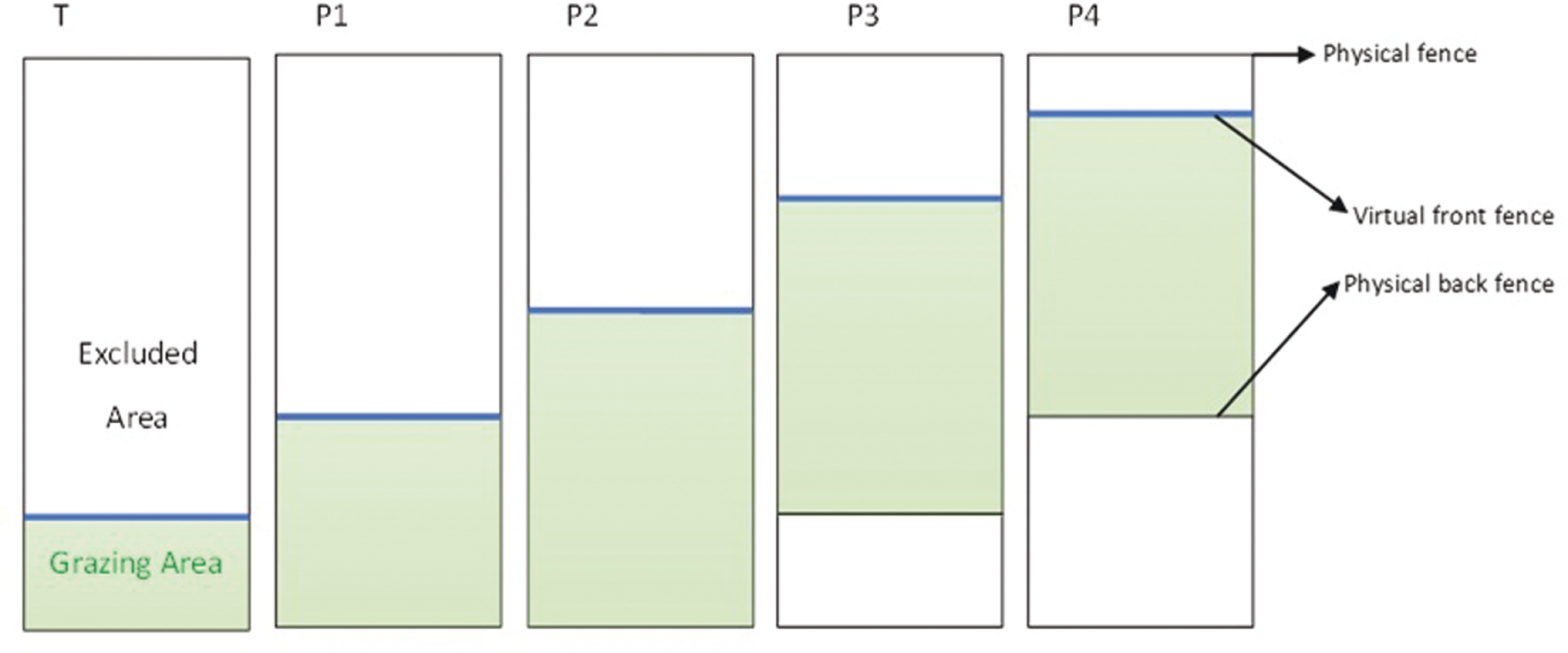
Illustration of the grazing regime during training (T) and four grazing periods (P1 to P4). Green zones represent the available grazing area of each period, delimited by virtual fences (blue lines) and electrical physical fences (black lines).

Twenty lactating Holstein-Friesian cows were included in the experiment. All cows were used to daily grazing using electric wire fences, but had no prior exposure to VF. For daily grazing, the animals were divided into four groups of five animals each: two younger groups of primiparous cows (**Y1** = mean ± SD: 2.8 ± 0.3 and **Y2** = 2.8 ± 0.3 yr, named together group **Y**, 195 ± 41 d from calving) and two older groups of multiparous cows (**O1** = 8 ± 3.0 yr old; **O2** = 7 ± 1.4, named together group **O**, 163 ± 84 d from calving). The cows were on pasture half-days, starting after the afternoon milking (⁓1600 hours local time) until milking the next morning (⁓0600 hours), resulting in one experimental day unit. Night grazing was preferred in order to avoid heat stress (Legrand et al., 2009). During grazing, the groups were kept in four separate paddocks (Figure 1). The paddocks were comparable in terms of botanical composition and forage yield (Table 1). For the rest of the day, the animals were housed all together in one group in a ventilated free-stall barn with cubicles and unrestricted access to a concrete outdoor area.

**Table 1.** Size of grazing areas, mean compressed grass height measured by a rising plate meter at the beginning and at the end of each period and botanical composition

Paddock^1^	Period^2^	Size, ha^3^	Mean grass height at the beginning of each period, mm	Mean grass height at the end of each period, mm	Dominant plant species
1	T	0.4	72	42	Lolium perenne Phleum pratense, Poa trivialis, Trifolium repens, Trifolium pratense
	P1	0.6	59	49.5	
	P2	0.8	61	53.3	
	P3	0.6	60.7	56.3	
	P4	0.6	53.3	41	
2	T	0.4	70	47	Lolium perenne, Phleum pratense, Poa trivialis, Trifolium repens, Trifolium pratense
	P1	0.6	60	50	
	P2	0.8	61.6	54.6	
	P3	0.6	59.3	56.3	
	P4	0.6	53	39	
3	T	0.4	72	41	Lolium perenne, Phleum pratense, Poa trivialis, Trifolium repens, Trifolium pratense
	P1	0.6	59.5	49.5	
	P2	0.8	63.3	53	
	P3	0.6	56.5	52.6	
	P4	0.6	54.6	43	
4	T	0.4	69	41	Lolium perenne, Poa pratensis, Trifolium repens, Taraxacum officinale
	P1	0.5	64	53.5	
	P2	0.7	70.3	52.6	
	P3	0.6	67.3	62.6	
	P4	0.6	69.6	52.6	

^1^Experimetal paddcoks.Paddock 1 and 2 were grazed by old groups while paddocks three and four were grazed by young groups.

^2^Grazing Periods.

^3^Size of each paddocks, for each grazing period.

### Sensors

All animals were equipped with a VF collar (Nofence AS, Batnfjordsøra, Norway, second version release) and an IceQube pedometer (Peacock Technology Ltd, Stirling, UK). The overall VF system functions the same as described in Aaser et al. (2022). To prevent the VF collars from triggering any stimuli (i.e., acoustic warning or electrical pulse) while the cows were inside the farm buildings, devices provided by the manufacturer (i.e., Nofence Shelter Beacon), were installed in the barn and in the milking parlor. These devices use Bluetooth communication to automatically disable the Global Navigation Satellite System (**GNSS**) receivers of the VF collars. During the experiment, collars collected 89,610 records. Each record contained the time-stamped GNSS position, the time-stamped GNSS acoustic warnings, electrical pulses, and the duration of each acoustic warning delivered by the collars. These data were transmitted via mobile networks and were then downloaded from the Nofence web platform. A three-axis accelerometer pedometer was set to the right rear leg of each cow, recording the standing time (including walking), lying time, and total step count performed by the cows within a 15-min interval. They had internal memory capacity to collect data for up to 200 d. Then, through the IceHub hardware (Peacock Technology Ltd, Stirling, UK), which provided the communication with the sensors, the data were exported as.csv files. A proper fit of the collar and pedometer was checked weekly to prevent the animals from experiencing skin damage such as abrasions or pressure marks.

### Experimental design

At the beginning of the trial, each group grazed a specific paddock fenced all around by physical wire fences for four half-days of acclimatization, during which the collars were worn but the VF was de-activated, followed by seven half-days of the training period (**T**). During the training, an electric wire fence was removed at one site of the paddock and a virtual boundary was set in its place. After 7 d, this virtual front fence was moved forward to provide new grazing areas for the cows (first period = **P1**). This procedure was repeated four times every 6 d, resulting in four periods of experimental treatment (**P1** to **P4**, Figure 1). Starting from P3, an additional electric wire fence was placed at the back of the paddock to prevent pasture damage to the already grazed area (Figure 1). Grass height was measured approximately every second day of each period with a semi-automated Rising Plate Meter (Grasshopper, G2 Sensor, TrueNorth Technologies, Shannon, Ireland) to ensure that enough forage was available for grazing (Table 1). According to these measurements, the estimated forage biomass available at the beginning of the grazing trial was 1.5 t DM ha^−1^. If an animal escaped from the virtual boundaries, we waited until it returned to the paddock on its own and did not guide it back.

### Milk yield and hair cortisol analysis

The milk yield of each cow was automatically recorded twice per day throughout the entire experiment by a 5 × 4 tandem-milking parlor (Lemmer-Fullwood AG, Gunzwil, Switzerland).

Analysis of hair cortisol concentration is a simple, noninvasive, and fast method to represent circulating long-term cortisol levels in dairy cattle as an indicator of stress (Tallo-Parra et al., 2015). Therefore, hair samples were collected on two sampling times: the first and last day of the experimental period. Samples were taken from the head of each cow by means of an electric blade—both times from the same area and with the same pre-cleaned blade. Forty samples in total were collected and then stored in a dry and dark place, to avoid any ultraviolet light contaminations. Cortisol concentration was measured in the regrown hair between the two cuttings according to Accorsi et al. (2008). This “shave-reshave” method ensured that sufficient cortisol was present in the regrown hairs for analysis (Heimbürge et al., 2019). In our case, 30 d was enough, considering that dairy cattle hair grows approximately 0.6 to 1 cm/mo (Schwertl et al., 2003).

### Data acquisition andprocessing

The collected data were processed using R software v. 4.2.2 (R core Team, 2021). Data gathered from VF collars were used to assess the differences in learning capacity between young and old cows. Target dependent variables were the total number and duration of acoustic warnings and the total number of electrical pulses. For the total number and duration of acoustic warnings, only data that were not followed by an electrical pulse were considered in order to investigate only those stimuli that induced the desired animal reaction (i.e., avoidance of the electrical pulse). Data on the duration of acoustic warnings were log-transformed to meet normality requirements. The success rate of the training period, defined as the total number of acoustic warnings not followed by an electrical pulse divided by the total number of acoustic warnings (Eftang et al., 2022), was calculated to describe the speed of the learning process to avoid the electrical pulses.

Pedometer data of standing time, lying time, and total step count were restricted to the time of the day when the animals were on pasture (i.e., from 1600 to 0600 hours) and summed on a daily basis.

Individual milk yields, for each experimental day and for the 15 d before and after the trial, were summed to obtain the daily milk yield (kg/d/cow). The hair cortisol content data were log-transformed to meet normality requirements.

### Statistical analyses

For each variable studied, repeated observations of a single animal over each experimental day in which VF was activated (i.e., from days 1 to 31), were accounted for. All data from day 8 were excluded from data analysis due to a malfunction of the system related to a GNSS inaccuracy.

Generalized mixed models were fitted with fixed effects of Age (*n* = two levels: O and Y), Period (*n* = five levels: T, P1 to P4), and day within each period (as a numeric value), and their two-way and three-way interactions. Animal data nested in groups (*n* = four groups replicated with five animals each) and the date of observation were included as random intercepts. For count variables (i.e., number of acoustic warnings, total step count, standing time, and lying time), a negative binomial likelihood distribution with log link function was used. For the number of electrical pulses, the negative binomial model did not converge because it contained too many zeros and a compound Poisson model was used. For the success rate, a binomial model was used. For mean warning duration, daily milk yield, and hair cortisol content a Gaussian likelihood distribution was used. In the model for daily milk yield, the THI and the lactation stage were included as additional covariates. For the latter, the non-significant three-way interaction was not included in the model.

Model parameters were estimated using the *“glmmTMB”* package (Brooks et al., 2017). Differences between age groups and within periods in mean values and temporal trends were tested using post hoc tests with Tukey adjustment using package *“emmeans”* (Searle et al., 1980). In addition, the R package *“DHARMa”* (Hartig, 2022) was used for the model diagnostic assumption. A *P*-value < 0.05 was considered significant. To better visualize and quantify the significance of *P* values, three significance levels are presented in the figures and tables: *P* < 0.05, *P* < 0.01, and *P* < 0.001.

## Results

### Response of animals to the VF system

**Figure 2.**
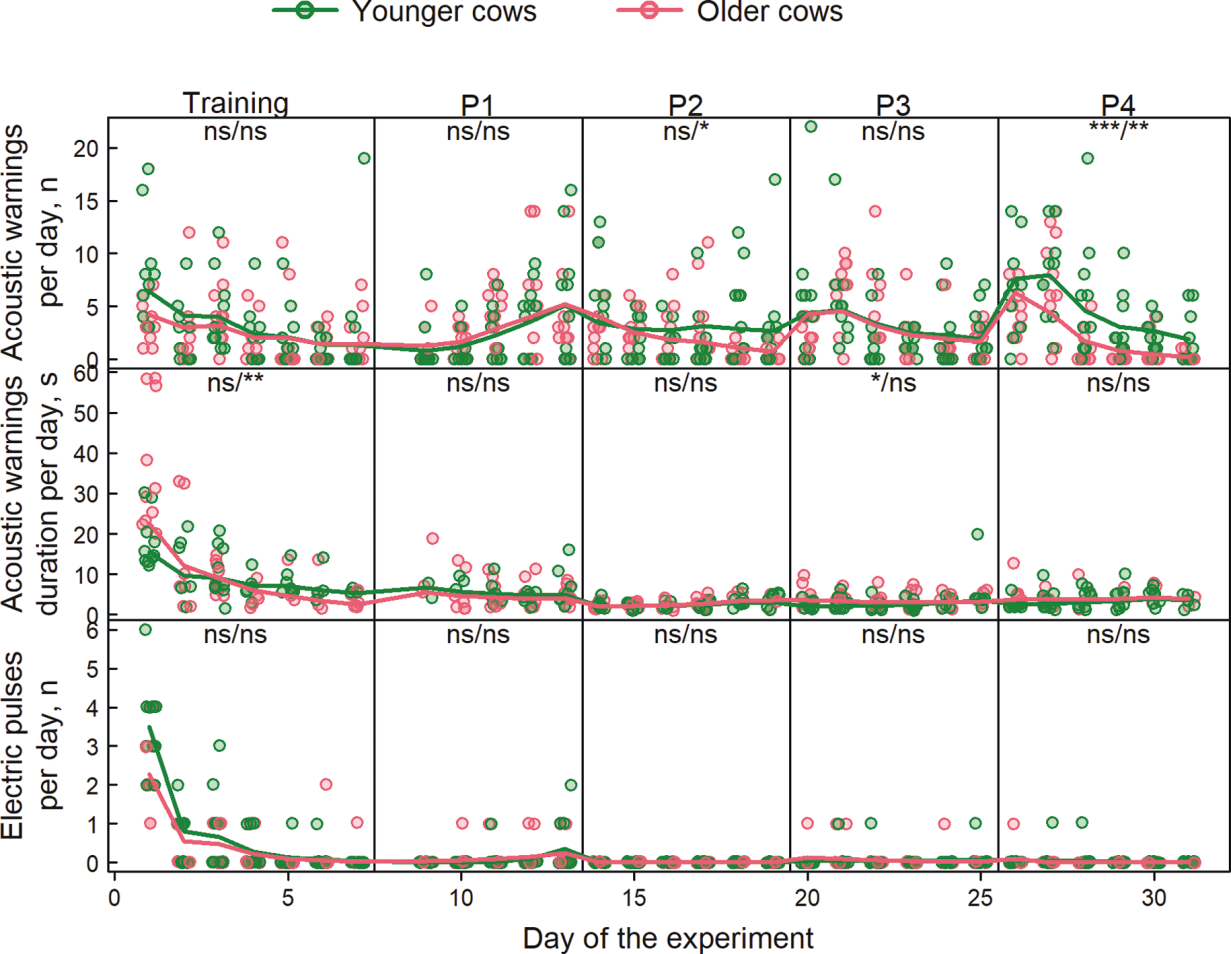
Daily number of acoustic warnings, duration of acoustic warnings, and electric pulses recorded by the virtual fencing collars during the 31 experimental days. Data points represent values for individual cows per day, colored lines are predicted average values from the fitted generalized linear mixed-effects models. Labels within each panel show the significance of the effects: the first label shows the significance of the age effect (younger vs. older animals) within each period; the second label shows the significance in the temporal trend within each period: ns = no significant difference, *P < 0.05, ** *P* < 0.01, and *** *P* < 0.001.

All animals were kept inside the defined grazing areas by VF most of the time during the experiment. In fact, escape events were only observed on the first and third days of the trial, when five cows in groups Y3 and one cow in Y4 escaped once. During the whole experimental trial, younger cows received a mean ± SD of 3.42 ± 4.10 acoustic warnings per day and per animal, while older ones received 2.58 ± 3.16 acoustic warnings per day and per animal (Figure 2). Because of the high variability, the age effect was not significant (Table 2). However, the triple interaction term (day within periods × age × periods) was significant, because younger cows received more acoustic warnings than older cows in P4, with a significant reduction in the number of acoustic warnings received per day within this period. This significant reduction per day was also observed for younger cows in period P2 (Figure 2). Also, acoustic warnings received per animal per day were affected by day within periods, periods, and their interaction (Table 2).

**Table 2.** Analysis of variance on the number of acoustic warnings, duration of acoustic warnings, and number of electrical pulses per day per cow, derived from generalized linear mixed-effect models

Source of variation^1^				
	df^2^	Acoustic warnings	Acoustic warning duration	Electric pulses
		Chisq^3^	Chisq^3^	Chisq^3^
Intercept	1	86.4^***^	15,581.3^***^	0
Day within periods	1	14.4^***^	0.9	0
Age	1	0.1	4.8^*^	0
Periods	4	40^***^	181^***^	46.5^***^
Day within periods × age	1	5.9^*^	3.8	0
Day within periods × periods	4	52.1^***^	49.9^***^	18.1^**^
Age × periods	4	3.1	5.7	3.6
Day within periods × age × periods	4	10.7^*^	5.4	2.3

^1^Sources of variation are day within periods, age, grazing period, and their interactions.

^2^Degrees of freedom.

^3^Chi-square values. Significance are indicated as * *P* < 0.05, ** *P* < 0.01, and *** *P* < 0.001.

Acoustic warning lasted on average (mean ± SD) 5.12 ± 4.92 and 6.38 ± 8.93 s/d, for younger cows and older cows, respectively (Figure 2). Over the entire experiment, age significantly affected the duration of acoustic warning per animal per day (Table 2). On the first day of training, acoustic warning lasted 18.0 ± 6.2 and 36.2 ± 15.7 s for younger and older cows, respectively, and rapidly decreased for both groups. Moreover, acoustic warnings lasted significantly longer for older cows in P3 as reflected by significant period effects and days within period × period interaction.

Younger cows received a mean ± SD of 0.21 ± 0.73 electrical pulses per day, while older ones received on mean ± SD of 0.54 ± 0.16 electrical pulses. Age did not affect the number of electrical pulses received by the cows. Periods and their interaction with the days within periods had a significant effect on the number of electrical pulses per animal per day, with the training having the highest values (Table 2). However, no significant differences between ages were detected (Figure 2).

**Figure 3.**
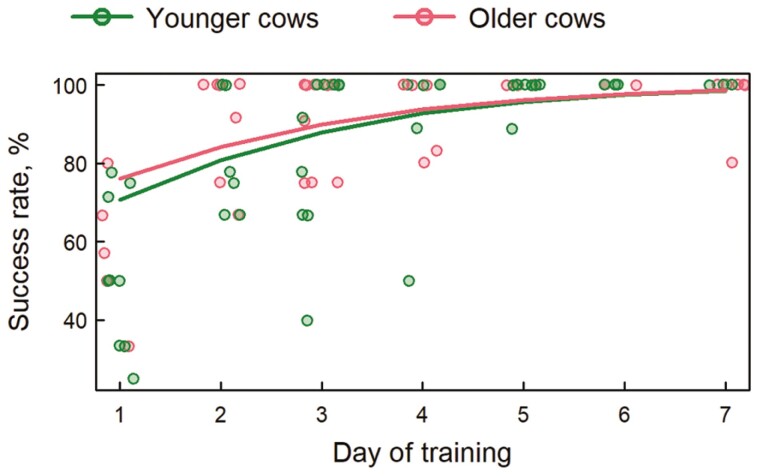
Daily success rate registered during the 7 d of the training. Data points represent values for individual cows per day, colored lines are predicted average values from the fitted generalized linear mixed-effects models.

On the first day of training, 49% ± 39 % (mean ± SD) of acoustic warnings for older cows and 47% ± 25 % for younger cows were successful (i.e., not followed by an electrical pulse). The success rate of audio tones rapidly increased during training for both ages and reached 100% ± 0 % and 98% ± 6 % on day 7 of the training period (Figure 3), for older cows and younger cows respectively. The success rate and its increase during training were not affected by age (Table 3).

**Table 3.** Analysis of variance on the success rate (i.e., ratio of acoustic warnings not followed by electrical pulses to the total number of acoustic warnings), derived from generalized linear mixed-effect model

Source of variation^1^		
		Success rate
	df^2^	Chisq^3^
Intercept	1	1.22
Age	1	0.31
Days of training	1	16.74^***^
Age × days of training	1	0.02

^1^Sources of variation are age, days of training, and their interaction.

^2^Degrees of freedom.

^3^Chi-square values. Significance are indicated as * *P* < 0.05, ** *P* < 0.01, and *** *P* < 0.001.

### Activity behavior

**Figure 4.**
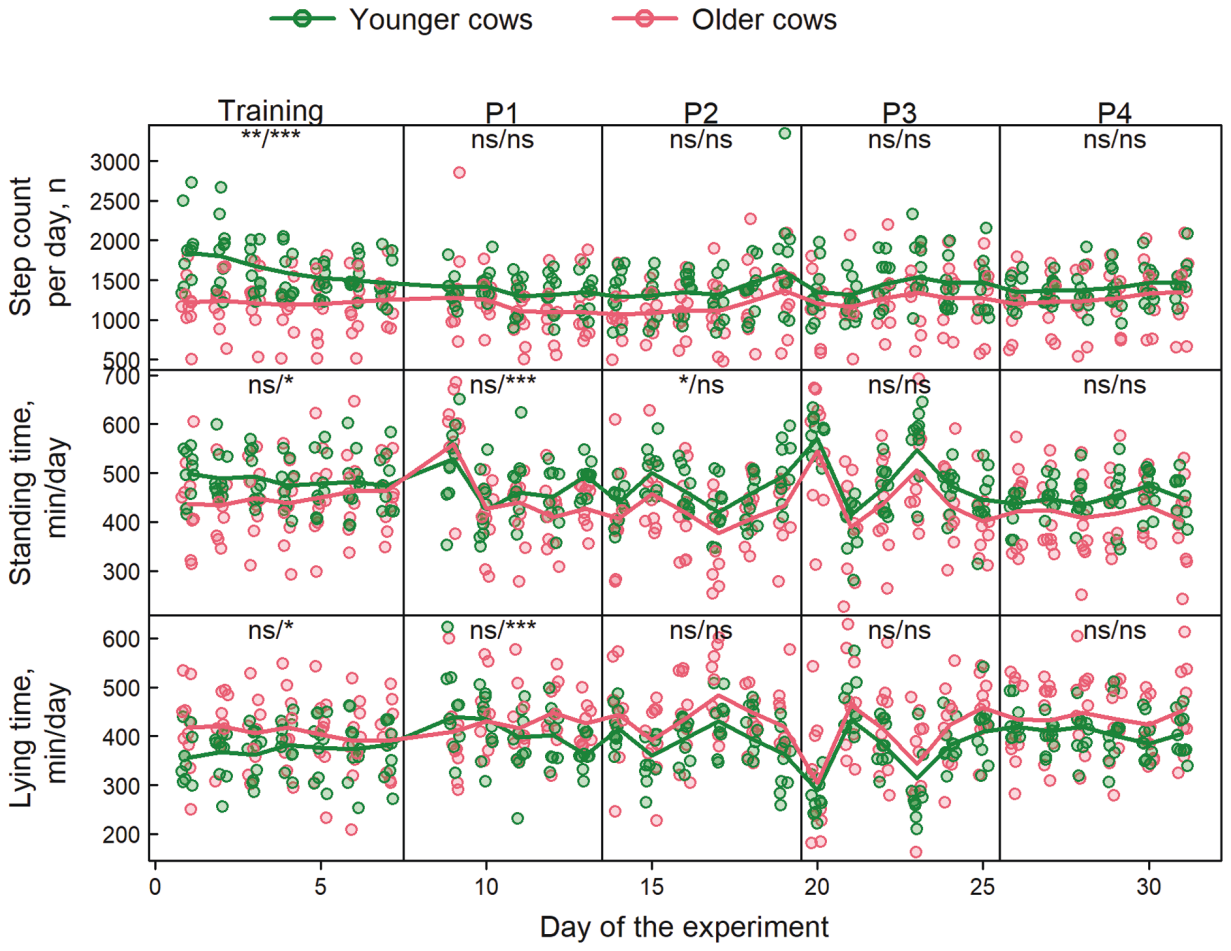
Daily number of steps, daily minutes spent in standing and lying position while at pasture, recorded by the pedometers during the 31 experimental days. Data points represent values for individual cows per day, colored lines are predicted average values from the fitted generalized linear mixed-effects models. Labels within each panel show the significance of effects: the first label shows the significance of the age effect (younger vs. older animals) within each period; the second label shows the significance in the temporal trend within each period: ns = no significant difference, * *P* < 0.05, ** *P* < 0.01, and *** *P* < 0.001.

During the entire experiment, the pedometer recorded 1,452 ± 336 steps (mean ± SD) per day for the younger cows and 1,214 ± 374 for the older ones. Daily step counts were significantly affected by age, periods, and their interaction, as well as by day within periods × periods interaction and the triple interaction (Table 4). This is because mean contrasts revealed a significant difference in daily step count between age groups during training (Figure 4). Thus, younger cows took more steps per day than older cows, with a significant reduction during the days of the training period.

**Table 4.** Analysis of variance on the number of steps, standing or walking time, and lying time per cow per day, derived from generalized linear mixed-effect models

Source of variation^1^				
	df^2^	Steps	Standing + walking time	Lying time
		Chisq^3^	Chisq^3^	Chisq^3^
Intercept	1	17,055.9^***^	32,215^***^	27,329.2^***^
Day within periods	1	2	1.7	0.1
Age	1	4.1^*^	1.2	0.6
Periods	4	38.6^***^	6.2	10.5^*^
Day within periods × age	1	0.4	7.5^**^	7.3^**^
Day within periods × periods	4	31^**^	3.4	4.8
Age × periods	4	46.9^***^	18.5^***^	18.2^**^
Day within periods × age × periods	4	15.8^**^	20^***^	19.3^***^

^1^Sources of variation are day within periods, age, grazing period, and their interaction.

^2^Degrees of freedom.

^3^Chi-square values. Significance are indicated as * *P* < 0.05, ** *P* < 0.01, and *** *P* < 0.001.

Younger cows stood slightly longer (471 ± 63 min/d) than older cows (437 ± 87 min/d), on average. However, time spent standing was not affected by age, periods, and day within periods, but it was affected by the interaction of the age with periods and days within periods, and by the triple interaction as well (Table 4). Thus, in P2 younger cows spent more time standing than older ones (Figure 4). Furthermore, older cows stood progressively more during T and less during P1.

While at pasture, older cows spent more time lying per day (421 ± 64 min) than younger cows (387 ± 83 min), but the overall effect of age was not significant. Periods, age × periods, and age × days within periods, as well as the triple interaction, had a significant effect on the lying time (Table 4). As for standing time, younger cows showed a different behavior during T than P1. Indeed, the time spent lying increased in younger cows during T, while it decreased during P1 (Figure 4).

### Milk yield and hair cortisol content

**Figure 5.**
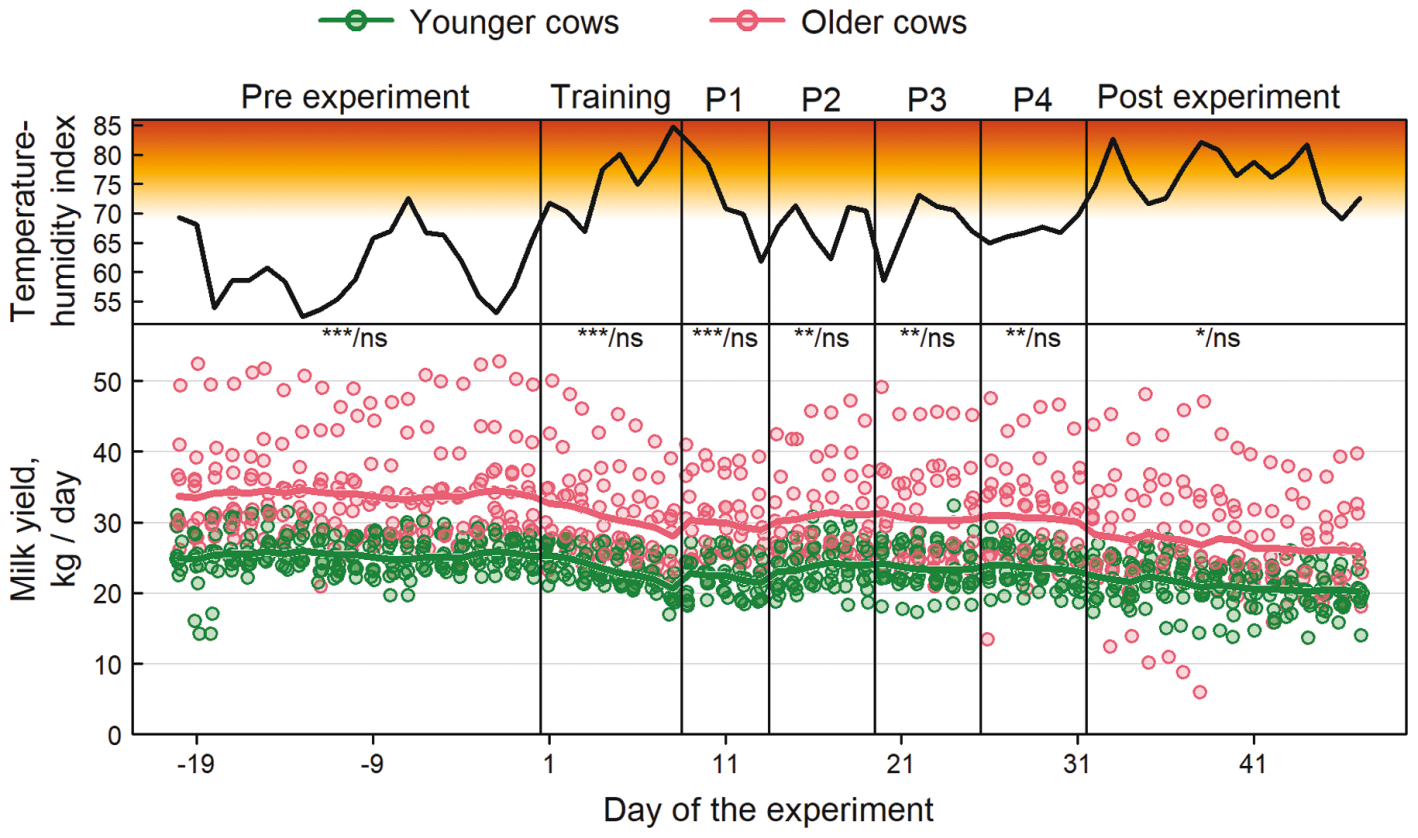
Total daily milk yield in the pre-experimental, experimental, and post-experimental periods. Data points represent values for individual cows per day, colored lines are predicted average values from the fitted generalized linear mixed-effects model. Red-scale represents the Temperature Humidity Index (THI) heat stress range, while black line presents the mean daily THI. Labels show the significance of differences: the first label shows the age effect within each period; the second label shows the significance of the temporal trend within each period: ns = no significant difference, * *P* < 0.05, ** *P* < 0.01, and *** *P* < 0.001.

Older cows produced significantly more milk per day than younger cows during the trial (mean ± SD: 30.86 ± 3.26 kg vs. 23.40 ± 7.36 kg) as well as in the pre- and post-experimental periods (mean ± SD: 33.94 ± 6.96 kg vs. 25.35 ± 2.74 kg and 27.08 ± 7.69 kg vs. 21.07 ± 3.02 kg; Figure 5). Milk yield was affected by age, periods, day within periods, and by the day within periods × periods and age × periods interaction, as well (Table 5). In addition, mean daily THI and Lactation stage affected the total milk yield (Table 5). Contrast between age groups did not reveal any difference in milk yield among days of each period of the VF treatment (Figure 5).

**Table 5.** Effect of age, grazing period, day within periods, Temperature Humidity Index (THI), and lactation stage on the total milk yield, derived from a generalized linear mixed-effect model

Source of variation^1^		Milk yield
	df^2^	Chisq^3^
Intercept	1	194.74^***^
Day within periods	1	9.85^**^
Age	1	11.56^***^
Periods	6	25.31^***^
THI	1	12.56^***^
Lactation stage	1	15.57^***^
Day within periods × age	1	0.13
Day within periods × periods	6	68.90^***^
Age × periods	6	38.62^**^

^1^Sources of variation are day within periods, age, grazing period (including pre-experiment and post-experiment), THI, lactation stage, and their interactions.

^2^Degrees of freedom.

^3^Chi-square values. Significance are indicated as * *P* < 0.05, ** *P* < 0.01, and *** *P* < 0.001.

Hair cortisol content averaged from 0.06 ± 0.05 (mean ± SD) and 0.12 ± 0.11 pc/mg (first-day samples) to 0.07 ± 0.01 and 0.09 ± 0.07 pc/mg (last-day samples), for younger cows and older cows respectively. Both the age and time of sampling, as well as their interaction did not significantly affect hair cortisol content (Table 6).

**Table 6.** Effect of age, sampling time, and their interaction on the hair cortisol content, derived from a generalized linear mixed-effect model.

Source of variation^1^		Hair cortisol content
	df^2^	Chisq^3^
Intercept	1	<2.2e-16 ^***^
Age	1	0.10
Sampling time	1	0.84
Age × sampling time	1	0.22

^1^Sources of variation are age, sampling time (beginning vs. end of the trial), day, and their interaction.

^2^Degrees of freedom.

^3^Chi-square values. Significance are indicated as * *P* < 0.05, ** *P* < 0.01, and *** *P* < 0.001.

## Discussion

Our study demonstrated that young and old lactating Holstein-Friesian cows learned to adapt to the VF system equally fast. Thus, cows learned to connect the acoustic warnings to a subsequent electrical pulse, irrespective of their age. As a result, no differences in the number of electrical pulses, stress level, activity behavior, and milk yields were observed between age groups. Therefore, younger cows showed a similar learning performance than older ones, contrary to our hypothesis. In fact, during days of training, no differences in the success rate were observed among age groups. Moreover, after the first 48 h of the trial, the average number of electrical pulses received per cow and day sharply reduced for both ages, resulting in strong increase in the success rate for both age groups with time (Eftang et al., 2022). In addition, cows did not receive any electrical pulses in P2, but reacted to acoustic warnings only. This constant decrease in electrical pulses was found also in previous studies on both lactating (Langworthy et al., 2021; Fuchs et al., 2024) and non-lactating (Lomax et al., 2019) cows. This is in contrast to our initial hypothesis and to Verdon and Rawnsley (2020), who concluded that older heifers learned faster than younger ones. However, this inconsistency may be due to the different experimental approaches of the studies (i.e., individual testing on non-adult animals in Verdon and Rawnsley). Furthermore, the animals of the present study were already familiar with electric wire fences on pasture, likely having a quick association with a new stimulus (i.e., acoustic warning; Verdon et al., 2020).

Younger cows received a high number of acoustic warnings in P4. This may be due to a possible forage depletion on pasture, which in turn might have led cows to move more to search for available grass, thus resulting in an increased number of acoustic warnings (Langworthy et al., 2021). However, the effects of forage shortage can be excluded since it was similar for all groups. In addition, mean daily steps in P4 showed no significant differences between the two age groups. Despite this, the average number of acoustic warnings obtained in this study was low and comparable to those obtained in other studies (Lomax et al., 2019; Aaser et al., 2022).

Acoustic warning duration gives important information about animals ability to understand the paired stimuli, because it is directly linked to the animal reaction at the virtual boundary zone. The warning duration is expected to decrease over time (Confessore et al., 2022b), become stable (Staahltoft et al., 2023) and increase again once the animals are conditioned and fully familiar with the acoustic warning. In the present study, the overall acoustic warning duration strongly decreased during the training period and stabilized in the following period for both age groups. There was a significant difference between age groups in the duration of the acoustic warnings during the training. However, in contrast to our hypothesis, we observed a faster reduction in warning duration for old rather than for young animals. Since the duration and the total number of acoustic warnings during the training were the same for both age groups, a possible explanation could be related to a difference in cow temperament (Tőzsér et al., 2003). Thus, during the first week of the trial, the older animals were likely more cautious and strictly avoided an electrical pulse, whereas the younger ones grazed close to the virtual boundary zone, thereby taking the risk of triggering an electric pulse. In addition, the significantly higher number of daily steps taken by the younger animals during training confirmed that they were more active.

It is well known that many factors can affect the lying behavior of grazing cows, including age (Sepúlveda-Varas et al., 2014). In our case, older animals, in absolute value, spent more time lying and less time standing or walking than younger ones throughout the trial. However, the differences between the two age groups were small and likely due to individual differences in activity levels. Since other studies did not find any difference in step counts between virtually and traditionally fenced cattle (Campbell et al., 2019; Hamidi et al., 2022; Fuchs et al. 2024) it is unlikely that the differences observed between age groups are caused by the VF treatment.

Milk yield was maintained during the exposure to VF system. In our 30-d study, old cattle produced significantly more milk than the young, as is commonly known in agricultural practice (Khan and Shook, 1996). In both age groups, there was a continuous decline in milk yield as expected for a progressing lactation stage. This decrease was linear from the pre-experiment period, throughout the experiment to the post-experiment period. Thus, there was no significant effect on milk yield neither when the animals first got in contact with the VF nor when they were adapting to a new virtual fence. Since most studies on VF in dairy systems have been conducted on either heifers or dry cows (Lomax et al., 2019; Colusso et al., 2020; McSweeney et al., 2020; Verdon and Rawnsley, 2020; Verdon et al., 2020), analysis of VF impacts for lactating dairy cows is still scarce, as well as its impact on milk yield. Specifically, Verdon et al. (2021) showed that milk yield did not differ between VF and electric fence strip-grazing management systems, but this was investigated for a short period of time (i.e., 10-d trial) which may be too short to detect a lasting change in milk yield due to VF. At the same time, similar results were found by Fuchs et al. (2024), when these management systems were compared over a longer period of time. It is well known that milk yield is impaired by high temperatures and humidity (Osei-Amponsah et al., 2020). In our case, there was some decrease in milk yield during the experiment, namely at the end of the training period, which could indicate a stress reaction in the animals. However, this variation was highly negatively correlated and well explained by THI values over the threshold (i.e., above 68; Pinto et al., 2020), registered in those days.

There was no increase in the cortisol level in either age group from the first day to the last day of the trial. These findings go along with various studies (Campbell et al., 2019; Confessore et al., 2022b; Hamidi et al., 2022) that found no relationship between cortisol content and VF management, suggesting that VF does not cause long-term stress in cattle, regardless of age.

## Conclusions

This study demonstrates that age has no significant effect on the adaptation of lactating dairy cows managed with a VF system. Our results highlight that the capacity to learn to adapt to a VF system does not decrease with the age of cows, at least in an agriculturally relevant age range. Animals, irrespectively of age, adapted to the system quickly within two to five half-days of grazing. Neither activity behavior nor milk yield and hair cortisol content revealed evidence of stress in the cows during the period studied and irrespective of their age. Thus, a mixed-age herd structure is not an obstacle to implementing VF. Consequently, the use of this technology provides an opportunity for the intensive dairy system to promote the use of grazing, improving the use of pasture resources, and may also reduce labor. Further investigations are needed to determine whether the efficiency of VF implementation, as well as the animal interaction with VF are affected by either available grass biomass or forage quality.

## Abbreviations

dB: decibel
GNSS: global navigation satellite system
O: old group
P1, P2, P3, P4: periods of experimental treatment
T: training period
THI: temperature humidity index
VF: virtual fencing
Y: young group
